# SOCIAL NETWORKING SITE USE AND MENTAL HEALTH IN OLDER LGB CANADIANS IN THE CLSA

**DOI:** 10.1093/geroni/igae098.2487

**Published:** 2024-12-31

**Authors:** Alexandra Grady, Arne Stinchcombe

**Affiliations:** University of Ottawa, Ottawa, Ontario, Canada; University of Ottawa, Ottawa, Ontario, Canada

## Abstract

The Internet has historically been an important avenue for social connection between lesbian, gay, and bisexual (LGB) people. Social networking sites (SNS) may have positive mental health impacts for LGB people. Limited research has explored SNS use in older LGB adults. The purpose of this study was to examine SNS use by sexual orientation in a sample of heterosexual and LGB Canadians using data collected from Follow-up 1 of the Canadian Longitudinal Study on Aging (n=21,836; 583 LGB). Participants completed self-report measures of SNS use, depression symptoms (CESD-10), and loneliness (UCLA 3-item Loneliness Scale). After adjusting for age, gender, income, and education, a significant difference remained in the odds of LGB participants using SNS to make new friends (OR = 1.82, p<.001) and promote themselves or their work (OR = 1.34, p=.005) in comparison to heterosexual participants. In terms of mental health, the linear regression treating depression as the outcome variable showed an interaction between being LGB and using SNS to stay in touch with friends (B=-1.23, p=.012), such that using SNS to stay in touch with friends as an LGB person was associated with fewer depression symptoms. The linear regression treating loneliness as the outcome variable showed an interaction between being LGB and using SNS to make new friends (B=.44, p=.013), indicating that LGB people who use SNS to make new friends report more loneliness than heterosexual people who use SNS to make new friends. This study adds to the growing literature on how older LGB adults use SNS.

